# Auditable large language model curation of clinical notes refines glucagon-like peptide-1 initiation, persistence ascertainment, and compounding use beyond prescription records

**DOI:** 10.1093/biomethods/bpag035

**Published:** 2026-06-24

**Authors:** Gowtham Varma, Karthik Murugadoss, Mathews E Kurian, Jerin Varghese, A J Venkatakrishnan, Venky Soundararajan

**Affiliations:** nference Labs, Bangalore, India; nference, 1 Main Street, East Arcade Building, Cambridge, MA, United States; Metabolism Agentic Intelligence Atlas (MAIA), Cambridge, MA, United States; nference Labs, Bangalore, India; nference Labs, Bangalore, India; nference, 1 Main Street, East Arcade Building, Cambridge, MA, United States; Metabolism Agentic Intelligence Atlas (MAIA), Cambridge, MA, United States; nference Labs, Bangalore, India; nference, 1 Main Street, East Arcade Building, Cambridge, MA, United States; Metabolism Agentic Intelligence Atlas (MAIA), Cambridge, MA, United States

**Keywords:** semaglutide, tirzepatide, GLP-1, weight-loss, LLM, AI, peptide compounding, Wegovy, Zepbound

## Abstract

Real-world benefit from incretin-based therapies depends on actual treatment initiation and sustained exposure, yet structured prescription records may capture prescribing intent rather than confirmed use. Using a large federated, de-identified US EHR platform spanning over 29 million patients, we analyzed patterns of glucagon-like peptide-1 receptor agonists (GLP-1RA) initiation, persistence, and compounding from clinical notes using an evidence-linked large language model (LLM) pipeline evaluated against an independent physician-adjudicated extracted-event reference standard, achieving 98.4% accuracy for structural chunk triage and 88.2% accuracy for medication-status adjudication. Among 553 073 adults with at least one semaglutide (*n* = 376 697) or tirzepatide (*n* = 176 376) prescription and baseline weight availability, documented initiation within ±3 months of the first structured prescription was identified in 141 189 semaglutide patients and 62 040 tirzepatide patients. Among these patients with documented initiation, frictionless starts accounted for 70.1% of semaglutide and 77.9% of tirzepatide starts, initiation after documented friction accounted for 17.2% and 15.9%, and early interruption after initiation within 3 months accounted for 12.7% and 6.2%, respectively. We analyzed treatment persistence over an 18-month period in a subset of 69 976 patients with “frictionless initiation.” Clinical note-based and structured prescription-derived persistence yielded materially different trajectories within each drug (log-rank *P* **<** .001). A note-derived ascertainment approach showed that 42.3% and 43.1% of semaglutide and tirzepatide patients, respectively, continued therapy, while structured prescription-based ascertainment yielded higher persistence estimates of 55.4% for semaglutide and 58.3% for tirzepatide. Among the patients with a documented 3–18-month barrier or discontinuation event, insurance, cost barriers, perioperative holds and gastrointestinal adverse events were the top documented reasons. Compounded exposure was detected in 1696 frictionless initiators, with the first compounding signal occurring on or before the first structured prescription in 50.8% of semaglutide and 57.9% of tirzepatide patients. These findings show that AI curation of clinical notes can distinguish prescription intent from verified exposure. With the potential time-to-market confounding, semaglutide shows higher initiation and lower non-initiation than tirzepatide, with comparable note-confirmed persistence at 18 months. More broadly, these findings support integrative exposure ascertainment to strengthen real-world GLP-1 evidence generation, payer decision-making, safety surveillance, and strategies to improve sustained access and treatment continuity.

## Introduction

Glucagon-like peptide-1 receptor agonists (GLP-1RA) and dual GLP-1/glucose-dependent insulinotropic polypeptide receptor (GIPR) agonists have rapidly reshaped obesity and cardiometabolic care [1–4]. Semaglutide and tirzepatide can produce substantial weight loss and have moved from specialist use into mainstream practice [5, 6]. However, understanding safety and effectiveness in real-world clinical care requires an accurate understanding of treatment initiation and exposure patterns [7, 8].

Structured prescription records are essential for real-world evidence studies, but they may capture prescribing intent rather than verified medication use [7–9]. Most retrospective studies of anti-obesity medications rely on structured prescription or dispensing records [5, 10–12]. Those sources are essential, yet they do not fully resolve whether a patient actually started therapy, whether treatment was delayed by prior authorization or out-of-pocket cost, or why a medication was paused soon after initiation [13–15]. Primary medication non-adherence is common across therapeutic areas and has also been documented for anti-obesity medications in real-world studies [13, 16].

Clinically relevant exposure details often appear in free-text documentation, including progress notes, patient messages, refill communications, and medication reconciliation narratives. Large language models (LLMs) can unlock these details at scale, but for clinical research they must be auditable, schema-constrained and linked back to source evidence [17–19]. A further source of ambiguity in routine care is the use of compounded GLP-1 formulations and these ambiguities are especially relevant for GLP-1 therapies, where access barriers, medication shortages, and compounded formulations can disrupt conventional prescription-based exposure dating [20, 21]. In these patients, local structured prescribing may not capture the earliest clinically relevant evidence of exposure, particularly when therapy is initiated outside the recording health system, documented through reconciliation, or discussed in narrative before formal local prescribing appears. This creates a practical exposure-dating problem for real-world analyses that rely on first captured prescription as the time of treatment initiation [7–9].

In this study, we leverage a large federated de-identified electronic health records (EHRs) platform and an LLM-based clinical note curation pipeline to systematically capture treatment initiation, persistence, and compounding exposure directly from narrative documentation. We quantify note-confirmed initiation, identify barriers to starting and sustaining therapy, and compare note-derived versus prescription-based estimates of persistence and exposure.

## Materials and methods

### Study design and data source

We performed a retrospective observational study using de-identified longitudinal EHR through the nSights Federated Clinical Analytics Platform. The platform contains structured EHR fields, including prescriptions, diagnoses, laboratory measurements, anthropometric measurements, and provider specialty information, as well as de-identified free-text clinical notes from participating US academic medical centers and affiliated health systems. All analyses were performed within the governed federated analytic environment. All EHR data used in this study were de-identified by expert determination in accordance with the Health Insurance Portability and Accountability Act (HIPAA) Privacy Rule. The authors did not access identifiable private health information. Under the applicable institutional governance framework, the use of expert-determined de-identified data for this retrospective analysis was not considered human-subjects research requiring institutional review board approval.

### Cohort definition

Eligible patients were aged 18 years or older and had at least one qualifying semaglutide or tirzepatide prescription. The index date was the timestamp of the first qualifying prescription for that molecule (see Figure S1 for index date spread, see online supplementary material for a color version of this figure). To ensure baseline anthropometric context, patients were required to have at least one weight measurement from 3 months before through 1 day after the index prescription.

### LLM extraction pipeline

We used GPT-OSS-20B served through vLLM in a Python-based inference pipeline. Inference used a low-temperature setting (temperature 0.1) and top_k = 1 with schema-constrained structured output to reduce stochastic variability and enforce valid JSON. These settings were selected a priori to prioritize low-variance, schema-valid outputs rather than generative diversity. We therefore describe this configuration as low-temperature and reproducibility-oriented rather than strictly deterministic. Model performance metrics reported in this study therefore correspond to this fixed inference configuration. For each de-identified note chunk, the model extracted (i) structural chunk type, distinguishing narrative text from medication-list content; (ii) medication status, categorized as taking, not started, or not taking; (iii) explicitly documented status context, including barriers, interruption reasons, or compounding-related details when present; (iv) a short GLP-1-specific summary; and (v) evidence pointers linking the extraction to supporting source text within the governed analytic environment.

Inference was performed within the governed de-identified analytic environment for each participating US center. The pipeline did not transmit identifiable clinical notes to external public model APIs. Model inputs consisted of de-identified note chunks, and downstream analyses used schema-constrained derived outputs rather than raw identifiable clinical text. Cross-site analyses were conducted using de-identified derived outputs and aggregate summaries under the applicable data-use and platform governance framework.

### Phenotype definitions and decision hierarchy

Patients were assigned one mutually exclusive phenotype within the ±3-month note window using a deterministic hierarchy applied to the extracted evidence. “Frictionless initiation” required explicit narrative evidence of active use with no documented holding, stopping or failure to start. “Explicit non-initiation” required explicit evidence that the medication was never started and no evidence of taking. “Interrupted” required evidence that the medication had been taken and then held, stopped or discontinued. “Started after barrier” required an initial not-start signal followed by “taking” within the window. “Mixed or other” captured contradictory or complex patterns not meeting those definitions. “No documentation” was assigned when no qualifying narrative evidence of GLP-1 status was found.

### Validation

Validation was performed at the extracted-event level because the LLM pipeline operated on individual note chunks and medication-status extractions before deterministic patient-level phenotype assignment. The validation sample was drawn from GLP-1-related extracted events within the ±3-month index window and was designed to evaluate the two model decisions used in downstream phenotyping: structural chunk triage and medication-status adjudication.

We used a two-dimensional stratified sampling design based on model-assigned chunk type and medication status. The initial sampling matrix crossed four model-identified chunk types, namely patient narrative, medication list, assessment/plan, and history, with three medication-status classes: taking, not taking, and not started. This yielded 12 possible chunk-status strata. To reduce dominance by common taking events and ensure adequate representation of rare but clinically important documentation patterns, we targeted a maximum of 30 extracted events per stratum. Nine strata reached the target of 30 events each, while three history-related strata were less frequent in the extracted corpus and contributed 45 available events in total, yielding 315 manually reviewed extracted events. Two independent physician annotators reviewed each sampled event. Annotators adjudicated (i) structural source type, using a medication-list versus narrative-text distinction, and (ii) medication status, using the narrative-only evidence policy shown in Figure S2 (see online supplementary material for a color version of this figure). A composite reference standard was created from the reviewed set, and inter-rater reliability was summarized using Krippendorff’s alpha. Structural chunk triage was evaluated across all 315 reviewed events. Structurally verified medication-list events were extracted events that physician annotators confirmed came from auto-populated medication-list sections rather than clinician narrative text. These entries were included in the structural chunk triage evaluation but did not provide sufficient narrative context for medication-status adjudication under the narrative-evidence policy. They were therefore excluded from the medication-status performance denominator, leaving 221 physician-confirmed narrative-text events for medication-status adjudication.

### Narrative note selection and exclusion of medication lists

All clinical notes authored within ±3 months of the index prescription were eligible for review. The extraction target was clinician narrative documentation, including history of present illness, assessment and plan text and patient messaging narratives when preserved as note text. Auto-populated medication-list content was explicitly excluded from positive medication-status adjudication and downstream phenotype assignment when it appeared without accompanying clinician narrative context, so that active medication-list entries alone could not be misclassified as proof of medication use.

### Surfacing reasons for interruptions and discontinuation

The following definitions were used to group the reasons into meaningful buckets for summarization. Gastrointestinal adverse effects included drug-attributed GI symptoms such as nausea, vomiting, diarrhea, constipation, abdominal pain, and reflux. Other adverse effects included non-GI drug-attributed events such as injection-site reactions, palpitations, headache, fatigue, dizziness, rash, hair loss, or gallstones. Inadequate response indicated insufficient or plateaued weight or glycemic response. Insurance and cost barriers included payer-driven and affordability barriers such as prior authorization, denials, formulary exclusion, step therapy, copay, or out-of-pocket cost. Pharmacy and dispensing issues indicated fulfillment failures at the pharmacy level, including mail-order delays, failed fills, or dispensing errors not attributable to manufacturer supply. Manufacturer shortage indicated national shortage, backorder, or unavailability. Perioperative hold indicated a peri-procedural pause, including surgery, bariatric surgery, endoscopy, or anesthesia. Medical contraindication indicated physiology- or safety-driven clinical reasons for stopping or not starting therapy, including contraindication, pregnancy, pancreatitis, medullary thyroid cancer risk, gastroparesis, hypoglycemia, renal dysfunction, or hepatic dysfunction. Patient preference indicated a patient-initiated decision, including refusal, hesitancy, or preference for lifestyle modification. Other/unspecified indicated a documented barrier event in which reason text was absent or did not match any named category.

### Treatment persistence profiles

Longitudinal persistence and compounding analyses were conducted in a subset of participating health systems with continuous 3–18-month follow-up, consistent note availability, and stable documentation density required for reliable temporal alignment of narrative and structured data. This restriction was applied a priori to minimize bias from incomplete downstream capture and to ensure note-driven treatment status could be meaningfully interpreted over time. The resulting subcohort preserved the relative distribution of semaglutide and tirzepatide exposure observed in the broader federated population. Persistence was derived using an LLM-based clinical note abstraction approach that classified medication-status documentation over time. This analysis was restricted to 69 976 subset; results should therefore be interpreted as persistence analysis nested within the broader initiation-patterns study.

### Surfacing note-evident compounding GLP1 exposure and outcomes

For each of the frictionless initiators, we screened the LLM-generated summaries to identify the use of compounded formulations of semaglutide and tirzepatide such as off-brand or generic GLP-1 product names, non-standard pharmaceutical forms or GLP-1 coformulated with adjuvants characteristic of compounded vials (cyanocobalamin/B12, niacinamide, glycine, etc.) or nontraditional sourcing (telehealth, med-spa, weight-loss or wellness clinic, online or mail-order pharmacy, cash-pay, and outside provider). A patient was classified as having compounded GLP-1 exposure when active use was specified within the same context.

### Treatment persistence, dose titration, and trends during 3–18-month follow-up

Persistence was estimated under two complementary endpoint definitions applied to a frictionless initiator cohort, with each patient’s time origin set to the first clinical note confirming they were taking the index drug within ±3 months of their first corresponding prescription. The notes-based endpoint extracted post-anchor medication-status mentions from longitudinal clinical notes using the same LLM-based exposure-ascertainment pipeline and processed them through a deterministic state machine. A discontinuation event was recorded when an explicit not-taking statement was not followed within 60 days by a subsequent taking note that recovered the apparent interruption (the “grace period”; intended to absorb transient documentation gaps and brief, recovered interruptions without misclassifying them as terminal discontinuation). Conversely, to prevent a patient with only a single index-time taking mention and no further narrative evidence from being silently counted as persistent, an active-use claim required at least one taking note within the 180 days preceding the censor date (the “reaffirming-taking rule”). Patients with ongoing reaffirming notes but no qualifying discontinuation were censored at the timestamp of their last affirming note of being on the medication. The prescription-based endpoint reanalyzed the same cohort using structured pharmacy records and triggered a discontinuation when the silence interval following the last observed fill exceeded 180 days, after crediting each fill with 60 days of supply and a 60-day initial grace from the index. Both endpoints shared an 18-month follow-up horizon. A clinical note is a point-in-time observation as it certifies that the patient was taking the drug at the moment of that encounter, and nothing about what happens between encounters. A pharmacy fill is a forward-looking observation: the days-supply field on the fill record is an explicit, prospective claim that the patient has medication on hand through the fill date plus the supply window. Both endpoints shared an 18-month follow-up horizon. Persistence was estimated by Kaplan–Meier with Greenwood 95% confidence intervals, and within-drug agreement between the two endpoints was evaluated by Mantel–Haenszel-stratified log-rank tests.

### Statistical analysis

We summarized baseline characteristics, note-derived initiation phenotypes, documented barrier or discontinuation reasons, and clinical note counts separately for semaglutide and tirzepatide cohorts. Baseline between-cohort differences were described using standardized mean differences rather than null-hypothesis significance testing, given the large cohort size. For binary phenotype and reason-category comparisons, we reported absolute risk differences and unadjusted relative risks with Wald 95% confidence intervals. For the overall mutually exclusive initiation-phenotype distribution, we quantified association between drug cohort and phenotype using standardized mean difference (SMD) and Cramer’s V. Treatment persistence was estimated using Kaplan–Meier methods with Greenwood 95% confidence intervals, and within-drug differences between order-derived and note-derived persistence were assessed using log-rank tests. All analyses were descriptive; no causal adjustment or treatment-effect estimation was performed. Statistical analyses were performed in Python 3.10.2 using pandas 2.2.2, SciPy 1.31.0, and statsmodels 0.14.2.

### De-identification and HIPAA compliance certification

Prior to analysis, all EHR data were de-identified under an expert determination consistent with the HIPAA Privacy Rule (45 CFR §164.514(b)(1)). The de-identification methodology employed a multi-layered transformation approach to both structured and unstructured data fields [22]. In structured data, direct identifiers including patient names and precise geographic locations were excluded entirely, while indirect identifiers underwent specific transformations: patient identifiers, medical record numbers, and accession numbers were replaced with one-way cryptographic hashes using confidential salts to preserve linkage across patient encounters; all dates were shifted backward by patient-specific random offsets (1–31 days) to preserve temporal relationships while obscuring exact event timing; the ZIP codes were truncated to two-digit state-level resolution; and continuous variables including age, height, weight, and body mass index were thresholded to prevent identification of extreme values (for example, ages ≥89 years transformed to “89+” and body mass index [BMI] >40 transformed to “40+”).

In unstructured clinical text, notes were processed with an ensemble de-identification system before downstream LLM extraction. The system combined machine-learning-based named-entity detection with rule-based detectors to identify protected health information and other personally identifiable information, including names, contact information, medical record numbers, accession numbers, dates, locations, and identifier-like strings that achieved an estimated >99% recall for personally identifiable information (PII) detection, with detected identifiers replaced by plausible fictional surrogates in a participating health system [23]. The LLM inference workflow was applied after de-identification and operated on de-identified note chunks within the governed analytic environment. Evidence pointers linked model outputs back to source chunks for audit within that environment and were not a release of identifiable clinical notes.

### Code availability

The code used for this study is not publicly available because the analysis was implemented within the governed nSights federated clinical analytics environment and is coupled to restricted internal data infrastructure and de-identified patient-level EHR data that cannot be released. To support transparency and reproducibility, Supplementary Methods provide pseudocode for the major deterministic workflow components, including note selection, schema-constrained LLM extraction, medication-list exclusion, patient-level phenotype assignment, persistence-state construction, and compounding-exposure identification. A representative LLM prompt and structured-output schema excerpt are also provided. Requests for additional information about the analytic workflow should be directed to the corresponding author.

## Results

### Study population and cohort profile

The analytic cohorts included 376 697 adults with at least one semaglutide prescription and 176 376 with at least one tirzepatide prescription, each with a baseline weight recorded from 3 months before to 1 day after the first (“index”) prescription (Table 1). The semaglutide cohort was older on average (mean age 58.6 vs. 56.1 years; SMD 0.184) and had a higher baseline prevalence of type 2 diabetes (39.2% vs. 29.5%; χ^2^  *P* < .001). Baseline BMI was similar across cohorts (34.0 vs. 34.4 kg/m^2^; SMD −0.073). Among patients with captured provider specialty data, primary care involvement was nearly universal, followed by cardiology, endocrinology or diabetes, gastroenterology or hepatology, and obesity, nutrition or weight-management encounters, indicating broad multispecialty involvement during the initiation window (see Table 2).

**Table 1:** Baseline cohort and study period characteristics.

Characteristic	**Semaglutide (*n* = 376** **697)**	**Tirzepatide (*n* = 176** **376)**	SMD
**Age at index, mean (SD), years**	58.6 (13.6)	56.1 (13.6)	0.184
**Female sex, *n* (%)**	243 001 (64.7)	118 529 (67.5)	−0.059
**Type 2 diabetes before index, *n* (%)**	147 536 (39.2)	52 067 (29.5)	0.205
**HbA1c, mean (SD), %, among patients with baseline values (−90 d, +1 d)**	7.2 (1.7)	6.8 (1.6)	0.242
**HbA1c not observed within baseline window, *n* (%)**	237 319 (63.0)	115 350 (65.4)	−0.050
**HbA1c not observed within 180-day pre-index window, *n* (%)**	213 211 (56.6)	103 885 (58.9)	−0.047
**HbA1c not observed within 365-day pre-index window, *n* (%)**	187 972 (49.9)	90 305 (51.2)	−0.026
**BMI, mean (SD), kg/m², among patients with baseline values (−90 d, +1d)**	34.0 (5.4)	34.4 (5.5)	−0.073
**BMI not observed within baseline window, *n* (%)**	96 434 (25.6)	52 736 (29.9)	−0.096
**BMI not observed within 180-day pre-index window, *n* (%)**	84 380 (22.4)	43 212 (24.5)	−0.050
**BMI not observed within 365-day pre-index window, *n* (%)**	71 949 (19.1)	34 393 (19.5)	−0.010
**Either BMI or HbA1c not observed within baseline window, *n* (%)**	54 483 (14.5)	32 838 (18.6)	−0.109
**Calendar-period**	2018–2025	2022–2025	
**Clinical notes from ±90 days around index, mean ± SD**	29.6 ± 51.7	24.0 ± 39.5	
**GLP-1-related notes from ±90 days around index, mean ± SD**	2.9 ± 4.6	2.2 ± 3.0	

Values are shown as mean (SD) or *n* (%), unless otherwise indicated. The index date was defined as the first captured semaglutide or tirzepatide prescription. The baseline window was defined as 90 days before through 1 day after index. The 180-day and 365-day pre-index windows also included the index date plus 1 day to allow for measurements recorded contemporaneously with treatment initiation. Type 2 diabetes was ascertained from diagnosis records before index. SMDs compare semaglutide with tirzepatide; positive values indicate higher values or proportions in the semaglutide cohort. BMI, body mass index; HbA1c, glycated hemoglobin; SMD, standardized mean difference.

**Table 2:** Provider specialty groups observed in the 90-day initiation window.

Characteristic	**Semaglutide (*N* = 63** **149)**	**Tirzepatide (*N* = 30** **653)**
**Unique provider specialty groups, median (IQR)**	5 (4)	4 (3)
**Provider specialty groups observed, *n* (%)**		
**Primary care**	61 921 (98.1)	30 075 (98.1)
**Endocrinology or diabetes**	13 592 (21.5)	5620 (18.3)
**Cardiology**	22 990 (36.4)	9789 (31.9)
**Gastroenterology or hepatology**	10 533 (16.7)	4909 (16.0)
**Obesity, nutrition or weight management**	11 828 (18.7)	5215 (17.0)
**Other specialties**	60 723 (96.2)	29 006 (94.6)

Values are summarized among patients with provider specialty captured and specified in the 90-day initiation window; Records with unknown, not known and missing specialty values were excluded. For provider specialty group rows, patients could contribute to more than one group; therefore, percentages do not sum to 100%. Other defined specialties included hospital-based care, emergency medicine, anesthesiology, diagnostic specialties, nephrology, surgical subspecialties, neurology, pulmonary or critical care, oncology or hematology, transplant medicine, psychiatry or behavioral health, obstetrics and gynecology, dermatology, rheumatology, urology, infectious diseases, rehabilitation or pain medicine, pediatrics, pharmacy, nursing, social work and allied health specialties. IQR, interquartile range.

### Note-derived GLP-1 initiation phenotypes within 3 months of first prescription

**Figure 1:**
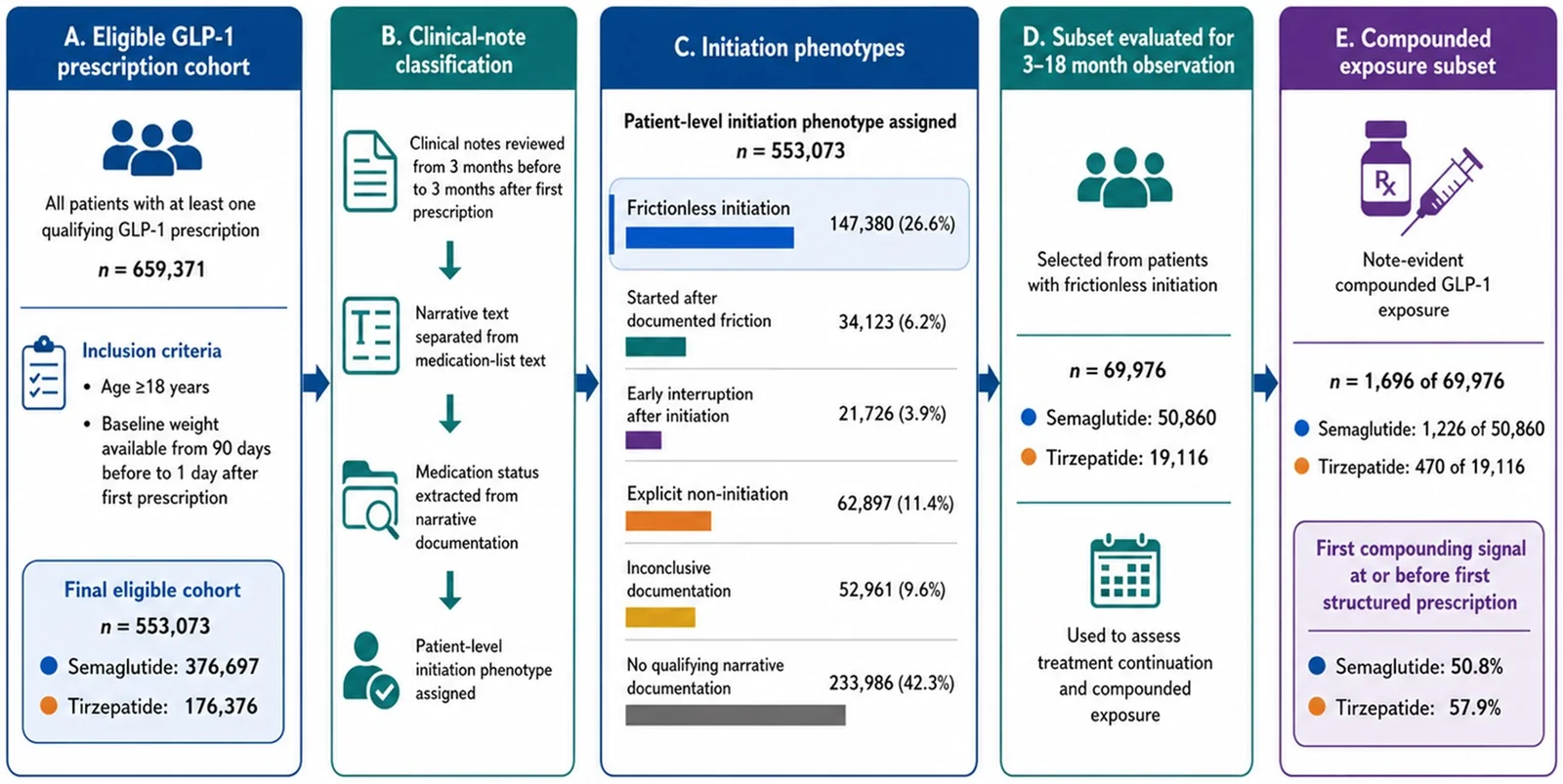
Study cohort, clinical-note classification, and longitudinal observation subset. (**A**) Eligible GLP-1 prescription cohort. (**B**) Clinical note classification. (**C**) Initiation phenotypes. (**D**) Subset evaluated for 3–18 month observation. (**E**) Compounded exposure subset. Adults with a first semaglutide or tirzepatide prescription and a baseline weight measurement were included. Clinical notes from 3 months before to 3 months after first prescription were reviewed to classify early GLP-1 initiation into mutually exclusive patient-level phenotypes. Patients with “frictionless initiation” were further evaluated for treatment persistence and compounded exposure during longitudinal follow-up. (Figure was generated using ChatGPT images 2.0 from author-specified study workflow instructions and was reviewed and edited by the authors for scientific accuracy.)

Among 553 073 adults with at least one semaglutide or tirzepatide prescription and baseline weight availability, clinical note-evidence within ±3 months of the first structured prescription was used to assign mutually exclusive documentation phenotypes of early GLP-1RA use (Table 3; definitions in Table S1). Documented initiation was identified in 141 189 semaglutide patients and 62 040 tirzepatide patients, representing 37.5% and 35.2% of the respective full prescription cohorts. Conversely, 235 508 semaglutide patients and 114 336 tirzepatide patients did not have note-confirmed initiation within the prespecified ±3-month narrative window. This latter group was not treated as confirmed non-use; it was further separated into no qualifying narrative documentation, “explicit non-initiation,” and inconclusive documentation. Among 141 189 patients with documented initiation, three distinct patterns were observed. Fig. 1 summarizes the federated phenotyping workflow and evidence-linked hierarchy used to assign patient-level initiation phenotypes. Cohort-level phenotype frequencies and between-cohort effect sizes are reported in Table 3. The largest group had “frictionless initiation,” defined as explicit narrative evidence of active use without documentation of holding, stopping, or failure to start. This pattern accounted for 99 033 semaglutide patients and 48 347 tirzepatide patients, corresponding to 70.1% and 77.9% of all note-confirmed starts, respectively. Initiation after documented friction, defined by an earlier non-initiation or access-barrier signal followed by later evidence of active use, included 24 247 semaglutide patients and 9876 tirzepatide patients, corresponding to 17.2% and 15.9% of documented starts. Early interruption after initiation included 17 909 semaglutide patients and 3817 tirzepatide patients, corresponding to 12.7% and 6.2% of documented starts. Among patients with early interruption, gastrointestinal adverse effects were the leading specific documented reason (Fig. 3; Table S2).

**Table 3:** Note-derived initiation phenotypes and between-cohort effect sizes.

Phenotype	Semaglutide	Tirzepatide	Absolute difference, pp (95% CI)	Relative risk (95% CI)
**Any documented initiation^a^**	141 189 (37.5%)	62 040 (35.2%)	+2.31 (2.03 to 2.58)	1.07 (1.06 to 1.07)
** Frictionless start**	99 033 (26.3%)	48 347 (27.4%)	−1.12 (−1.37 to −0.87)	0.96 (0.95 to 0.97)
** Started after friction**	24 247 (6.4%)	9876 (5.6%)	+0.84 (0.70 to 0.97)	1.15 (1.12 to 1.18)
** Early interruption after initiation**	17 909 (4.75%)	3817 (2.16%)	+2.59 (2.49 to 2.69)	2.20 (2.12 to 2.27)
**Explicit non-initiation**	40 818 (10.8%)	22 079 (12.5%)	−1.68 (−1.87 to −1.50)	0.87 (0.85 to 0.88)
**No documentation**	156 034 (41.4%)	77 952 (44.2%)	−2.77 (−3.06 to −2.49)	0.94 (0.93 to 0.94)
**Inconclusive sentiment**	38 656 (10.3%)	14 305 (8.1%)	+2.15 (1.99 to 2.31)	1.27 (1.24 to 1.29)

Absolute differences are calculated as semaglutide minus tirzepatide.

a Any documented initiation is a composite category and is not part of the mutually exclusive six-category phenotype hierarchy, which includes frictionless start, started after friction and early interruption after initiation groups.

**Figure 2:**
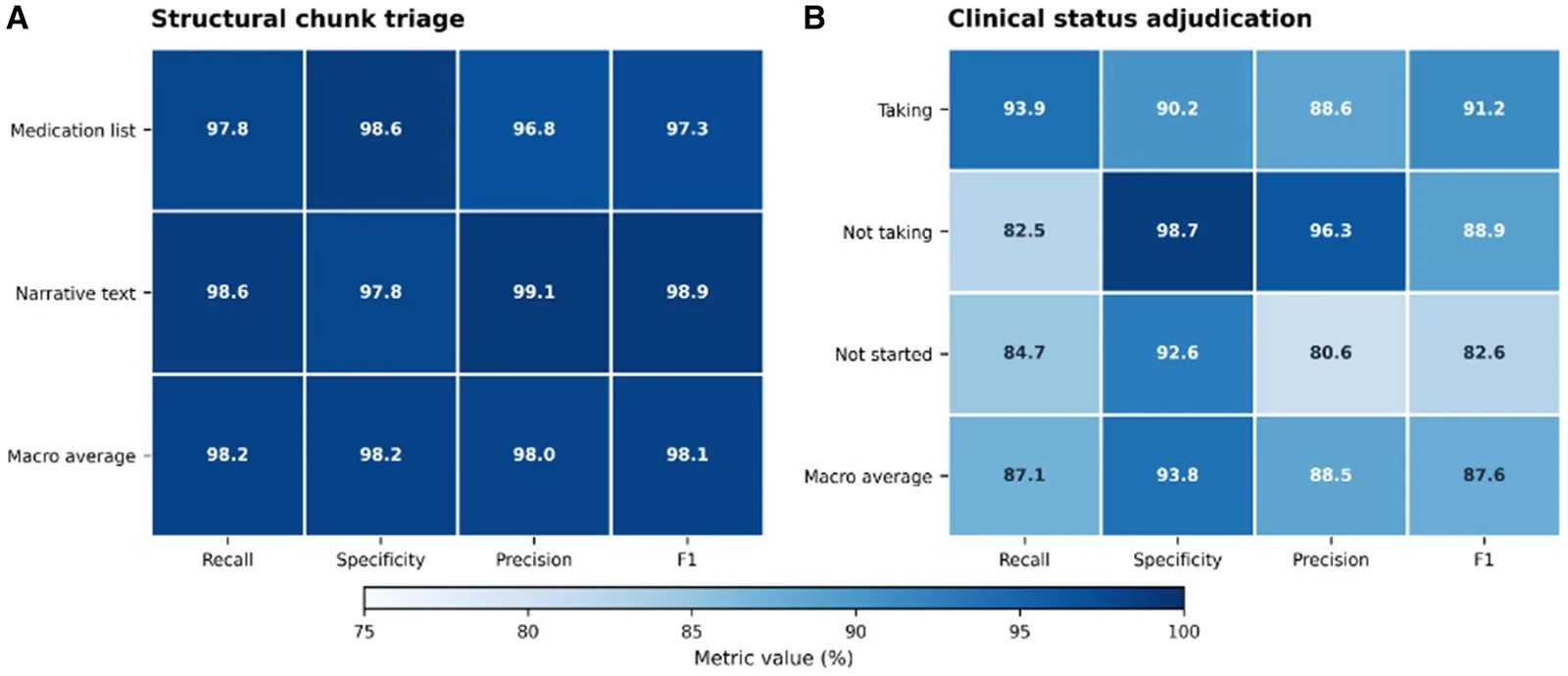
Class-level LLM validation performance. Heat maps summaries recall, specificity, precision, and F1 for (**A**) structural chunk triage and (**B**) clinical-status adjudication. The macro F1 was calculated across 315 reviewed extractions, while medication-intake ascertainment achieved 88.2% accuracy and 87.6% macro F1 across 221 reviewed events.

The schema-constrained LLM pipeline was evaluated against the physician-adjudicated reference standard for the two abstraction tasks used in phenotype assignment: structural chunk triage and medication-status adjudication from narrative text (see Fig. 2, Table 4, and Table S4). Structural triage achieved 98.4% accuracy and 98.1% macro F1 across 315 reviewed events. After excluding structurally verified medication-list events, medication-status adjudication achieved 88.2% accuracy and 87.6% macro F1 across 221 reviewed narrative events. Human inter-rater reliability was high for both tasks, with Krippendorff’s alpha of 0.93 for structural chunk triage and 0.81 for medication-status adjudication.

**Table 4:** Summary validation performance of the schema-constrained LLM pipeline.

Task	Reviewed events, *n*	Human IRR, Krippendorff’s alpha	Accuracy (%)	Macro F1 (%)
**Structural chunk triage**	315	0.933	98.4	98.1
**Clinical status adjudication**	221	0.805	88.2	87.6

Detailed class-level recall, specificity, precision, and F1 metrics are provided in Figure S1 (see online supplementary material for a color version of this figure) and Table S4. IRR, inter-rater reliability.

**Table 5:** Clinical interpretation of LLM-derived GLP-1 initiation, persistence, interruption, access-barrier, and compounded-exposure phenotypes supported by physician-adjudicated extracted-event validation.

Domain	Quantitative finding	Interpretation
**Verified initiation**	Note-confirmed initiation within ±3 months of first structured prescription was 37.5% for semaglutide and 35.2% for tirzepatide; absolute difference, +2.31 percentage points; 95% CI, 2.03 to 2.58; RR, 1.07 (Table 2). “frictionless initiation” was 26.3% for semaglutide and 27.4% for tirzepatide; RR, 0.96; 95% CI, 0.95 to 0.97 (Table 2).	Structured prescription records captured treatment intent but did not reliably establish early treatment use. Note-derived ascertainment identified modestly higher verified initiation for semaglutide, while tirzepatide showed a slightly higher frictionless-start phenotype. These differences should be interpreted descriptively given cohort differences in calendar time (Table S1), baseline comorbidities (Table 1), clinical encounter frequency, treating physician specialty mix, and documentation patterns reflected by the distribution of clinical notes including note burden (Table S5).
**Persistence ascertainment**	At 18 months, order-derived persistence was higher for tirzepatide than semaglutide, 58.3% versus 55.4%; log-rank χ² = 50.0; *P* < .001. However, by contrast, note-derived persistence was similar: 43.1% versus 42.3%; log-rank χ² = 1.3; *P* = .25 (Fig. 4)	Prescription-derived persistence and note-derived active-use persistence were not interchangeable. The apparent tirzepatide advantage observed using structured orders was not reproduced when persistence was defined by clinical-note-confirmed active use.
**Early interruption**	Early interruption after initiation was more frequent for semaglutide than tirzepatide: 4.75% versus 2.16%; absolute difference, +2.59 percentage points; 95% CI, 2.49 to 2.69; RR, 2.20; 95% CI, 2.12 to 2.27. Gastrointestinal adverse effects were documented in 13.6% of “interrupted” semaglutide patients and 9.0% of “interrupted” tirzepatide patients.	Early interruption represents a clinically important exposure phenotype that is not adequately captured by prescription records alone. The higher semaglutide interruption signal highlights the need to distinguish tolerability, titration, temporary holds, re-initiation, and persistence as distinct components of real-world exposure.
**Access and payer friction**	“Explicit non-initiation” was lower for semaglutide than tirzepatide: 10.8% versus 12.5%; absolute difference, −1.68 percentage points; 95% CI, −1.87 to −1.50; RR, 0.87; 95% CI, 0.85 to 0.88. Prior authorization or insurance was the leading named non-initiation reason, documented in 23.3% of semaglutide non-initiators and 25.4% of tirzepatide non-initiators.	A meaningful fraction of patients had explicit evidence of non-initiation despite a structured prescription. Access barriers, particularly prior authorization and insurance-related issues, were prominent contributors to discordance between prescribed and actual use.
**Compounded exposure**	Among patients with note-evident compounded exposure, the first compounding signal occurred on or before the first structured prescription in 50.8% of semaglutide and 57.9% of tirzepatide patients. The first signal occurred before or within 180 days after structured prescription in 90.1% and 95.7%, respectively.	Compounded GLP-1 exposure was frequently documented early and sometimes preceded structured prescription visibility. This creates implications for exposure dating, safety surveillance, and interpretation of treatment initiation in real-world studies.

The LLM curation pipeline was validated against independent human annotation, achieving 98.4% accuracy and 98.1% macro F1 for structural chunk triage, and 88.2% accuracy and 87.6% macro F1 for medication-status adjudication.

**Figure 3:**
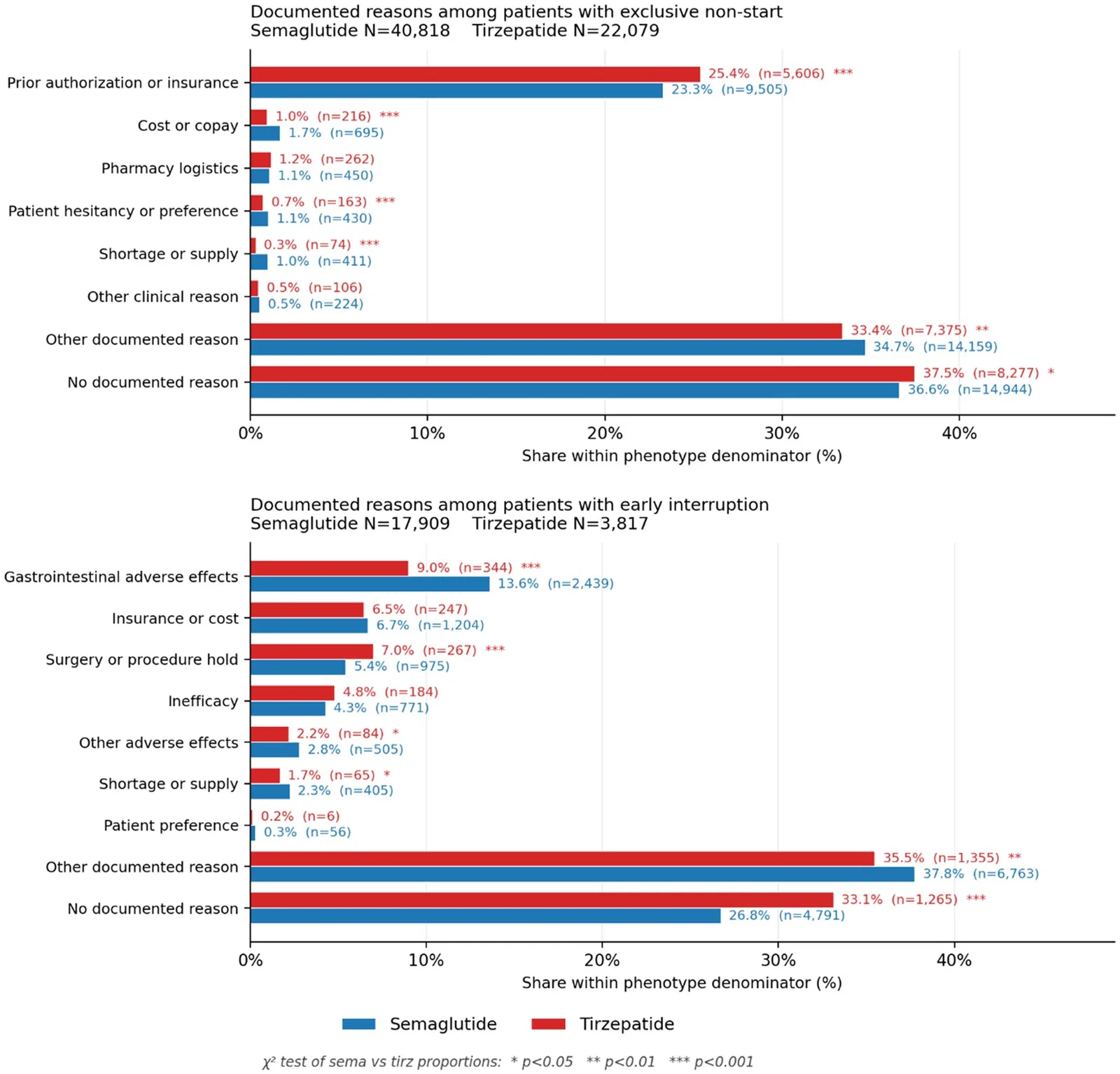
Documented reasons for non-initiation and early interruption (see methods for group definitions).

Among 349 844 patients without note-confirmed treatment start, the most common pattern was absence of qualifying narrative documentation (Table 5). No narrative evidence sufficient to assign initiation or interruption status was identified in 41.4% of semaglutide patients (*n* = 156 034) and 44.2% of tirzepatide patients (*n* = 77 952). “Explicit non-initiation” was documented in 10.8% of semaglutide patients (*n* = 40 818) and 12.5% of tirzepatide patients (*n* = 22 079), indicating that a meaningful subset had direct narrative evidence that therapy had not started despite the presence of a structured prescription. Prior authorization and insurance-related barriers were the most common specific documented reasons for non-initiation (Fig. 3; Table S3). Inconclusive documentation, including mixed or conflicting treatment-status signals that could not be assigned to another deterministic phenotype, was present in 10.3% of semaglutide patients (*n* = 38 656) and 8.1% of tirzepatide patients (*n* = 14 305).

### Treatment persistence estimates by ascertainment source

**Figure 4:**
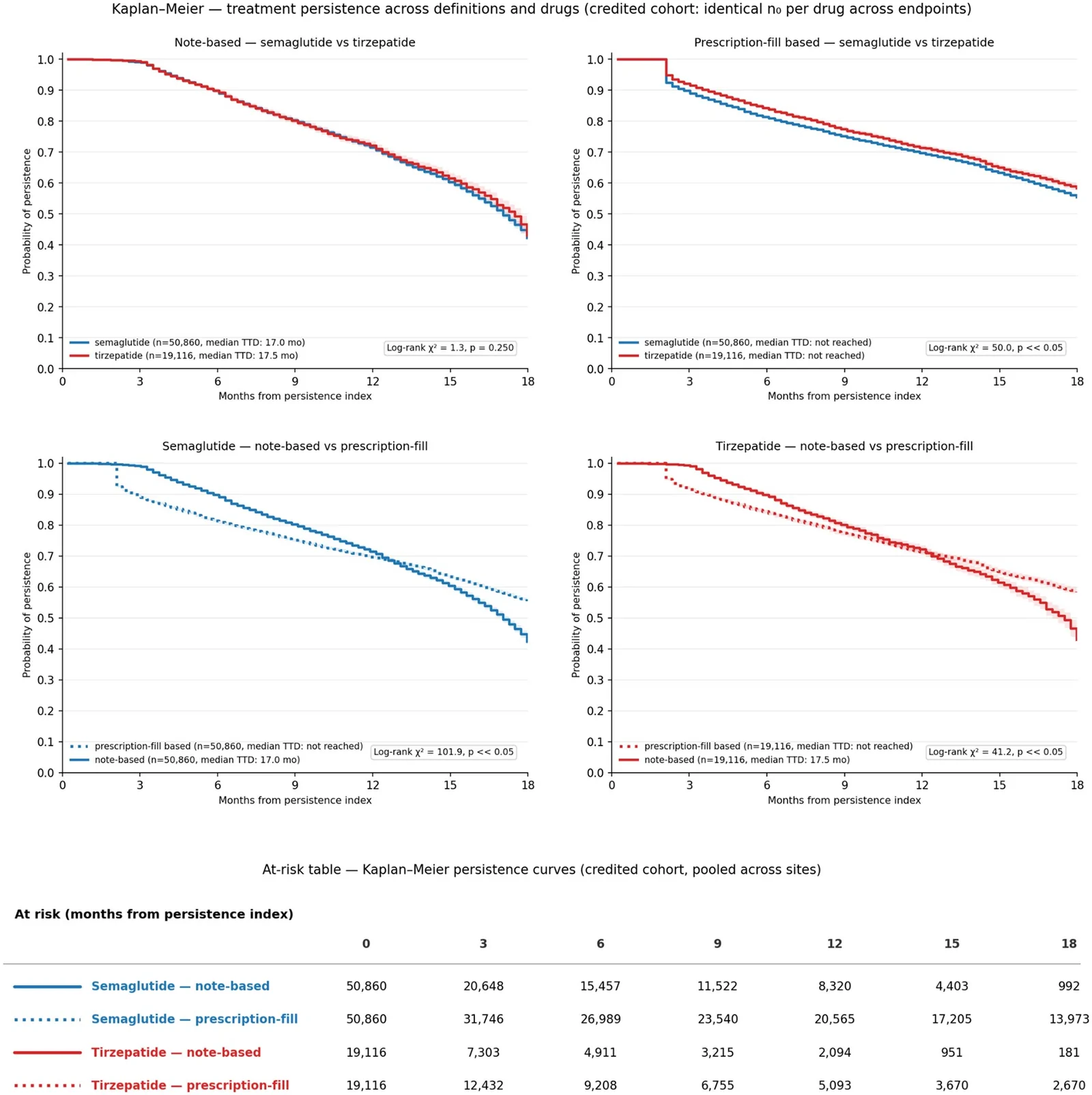
Treatment persistence of semaglutide and tirzepatide as defined from structured prescription orders and note-based LLM confirmation. Prescription-based persistence credited each prescription with a minimum 60-day supply and patients were censored at their last order plus 60 days if no subsequent order was observed within the next 180 days. Note-based persistence was estimated from longitudinal clinical-note evidence of active use and discontinuation using the rules described in the methods. Median time to discontinuation was not reached during observation using the prescription-based definition and was approximately 17.3 months using the note-based definition.

We estimated treatment persistence over 18 months in 69 976 patients with note-confirmed frictionless GLP-1 initiation (50 860 semaglutide and 19 116 tirzepatide) using two ascertainment approaches: structured prescription activity and clinical note-derived evidence of active use. The time origin (*T*_0_) was the date of the first note-confirmed GLP-1 exposure (Fig. 4). Persistence estimates differed materially depending on ascertainment source. Order-derived persistence was defined as continued structured GLP-1 prescription order activity within a 180-day gap window. Note-based persistence required clinician documentation of active use, allowing a 60-day interruption grace period and a 180-day reaffirming-taking rule (see the Methods section). For the note-based persistent analysis, absence of later GLP-1 narrative evidence was not counted as documented discontinuation. Instead, patients without sufficient subsequent narrative evidence of active use were censored according to the note-derived persistence ascertainment rules.

Within both the semaglutide and tirzepatide cohorts, the order-derived and note-derived curves diverged significantly (Fig. 4), within-drug comparisons between ascertainment approaches were statistically significant for both semaglutide and tirzepatide (log-rank *P* < .001 for within-drug comparison). At 18 months, persistence was lower using the note-derived approach than the order-derived approach for both drugs: 42.3% versus 55.4% for semaglutide and 43.1% versus 58.3% for tirzepatide. Median time to discontinuation was not reached using the order-derived definition, but was approximately 17.3 months using the note-derived definition for both drugs.

**Figure 5:**
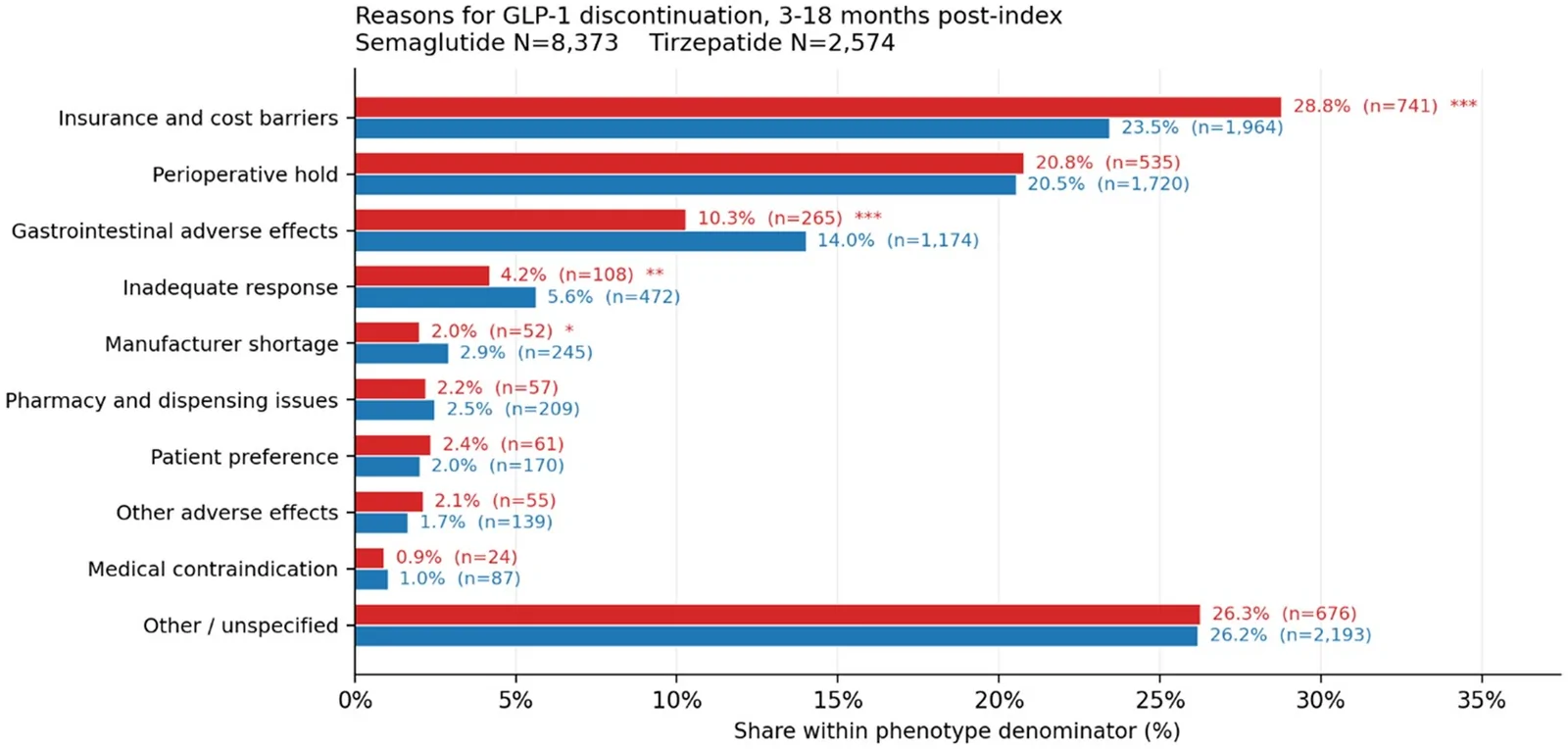
Reasons for discontinuation of semaglutide and tirzepatide (see methods for group definitions). The symbols * (statistically significant), ** (very significant) and *** (extremely significant) are explained further in the Methods.

Between-drug comparisons should be interpreted descriptively because semaglutide and tirzepatide cohorts differed by calendar time, indication mix, access environment, and clinical documentation patterns. Using the order-derived definition, tirzepatide showed significantly higher 18-month persistence than semaglutide (58.3% vs. 55.4%; log-rank χ^2^ = 50.0, *P* < .001). Using the note-derived definition, 18-month persistence was similar (43.1% vs. 42.3%; log-rank χ^2^ = 1.3, *P* = .25), although the full note-derived trajectories did not differ statistically between drugs. Among the patients with a documented 3–18-month barrier or discontinuation event, insurance, cost barriers, perioperative holds and gastrointestinal adverse events were the most commonly documented reasons (Fig. 5).

### Timing of first note-evident compounded GLP-1 exposure

**Figure 6:**
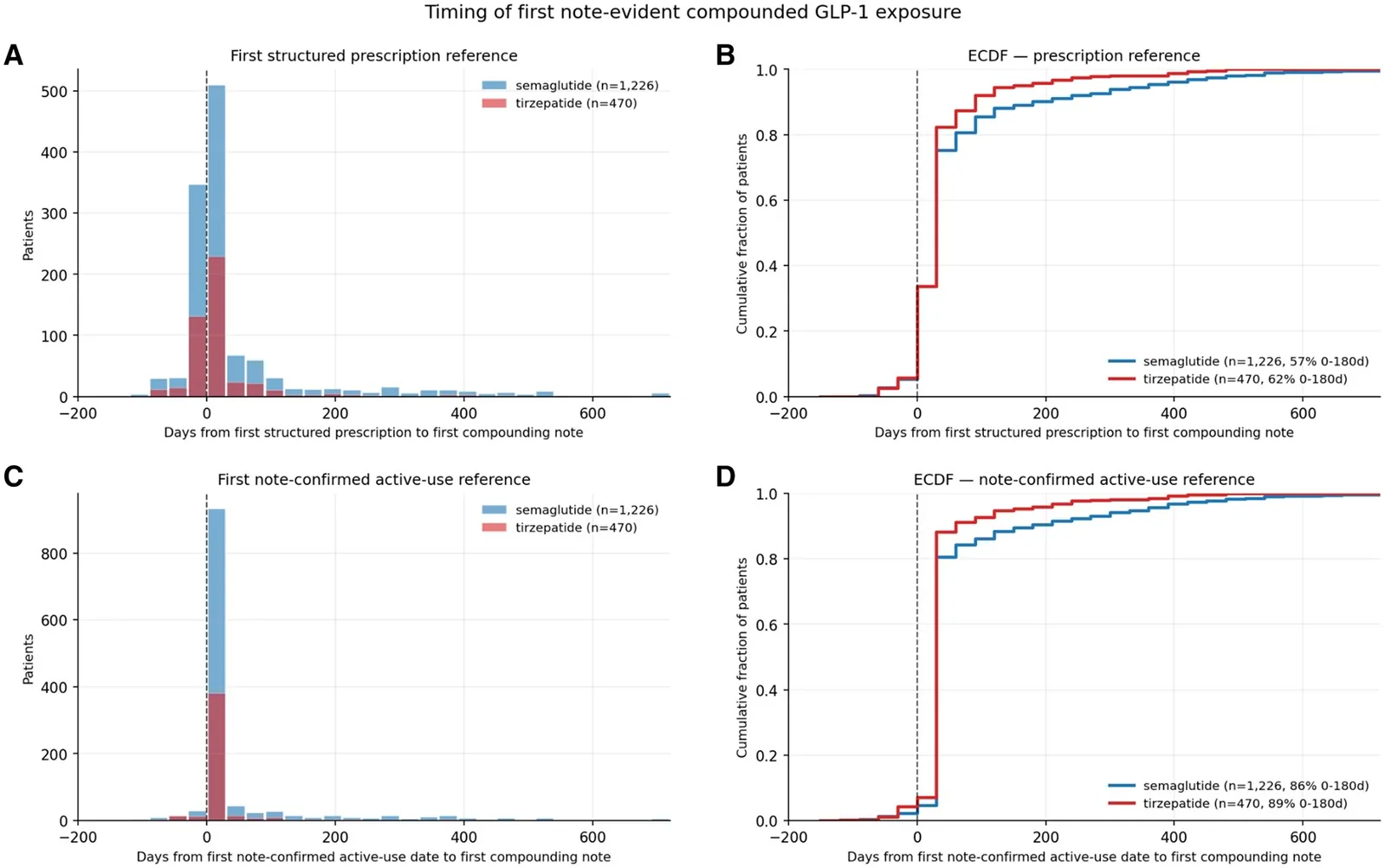
Timing of first note-evident compounded GLP-1 exposure relative to structured-prescription and note-confirmed treatment dates. (**A**, **B**) Time from the first captured structured prescription to the first clinical-note evidence of compounded semaglutide or tirzepatide exposure, shown as overlaid histograms (A) and empirical cumulative distribution functions (ECDFs; B). (**C**, **D**) Time from the first note-confirmed active-use date used for persistence follow-up to the first clinical-note evidence of compounded exposure, shown as overlaid histograms (C) and ECDFs (D). dashed vertical lines indicate day 0; negative values indicate that compounding was documented before the reference date, and positive values indicate documentation afterward. Relative to the first structured prescription, 33.6% of both semaglutide and tirzepatide patients had compounding documented before the prescription, while 56.5% and 62.1%, respectively, had first documentation from the prescription date through 180 days afterward. Relative to the note-confirmed active-use date, first compounding documentation was more tightly concentrated near treatment confirmation, with 85.7% of semaglutide and 88.9% of tirzepatide patients documented from day 0 through 180 days. Together, these distributions show that compounded GLP-1 exposure was usually documented early and could precede visibility in structured prescribing records.

Among 1696 patients with note-evident compounded semaglutide (1226) or tirzepatide (470) exposure, we evaluated the timing of the first compounding signal (note evidence) relative to two prespecified reference dates: the first captured structured prescription and the first note-confirmed active-use date used for persistence follow-up (Fig. 6). Relative to the first structured prescription, first compounding documentation was frequently observed before or on the date of the local structured prescription record. The first compounding signal occurred before the first structured prescription for 33.6% of both the semaglutide and tirzepatide cohorts and on the same date as the first structured prescription in 17.2% and 24.3%, respectively. Thus, the first compounding signal occurred on or before the first structured prescription date in 50.8% of semaglutide and 57.9% of tirzepatide patients. This indicates that, in many compound-exposed patients, the first local structured prescription may not represent the earliest clinically documented GLP-1 exposure. An additional 39.3% of semaglutide and 37.8% of tirzepatide patients had their first compounding signal after the prescription date but within 180 days. Overall, 90.1% of semaglutide and 95.7% of tirzepatide patients with note-evident compounding had their first compounding signal either before the structured prescription or within 180 days after it.

When timing was instead measured relative to the first note-confirmed active-use date, first compounding documentation was more tightly concentrated around the treatment confirmation date (Fig. 6C and D). First compounding documentation occurred between the confirmed active-use date through 180 days afterward in 85.7% of semaglutide and 88.9% of tirzepatide patients, while only 4.6% and 7.0%, respectively, had compounding evidence before the active-use date. Together, these findings suggest that compounded GLP-1 exposure is often documented early and can precede visibility in structured prescription records, supporting combined use of structured prescription data and note-derived evidence when defining treatment timing in real-world studies.

## Discussion

This study provides a large-scale, real-world view of how GLP-1 receptor agonists are initiated and used in routine clinical care, and it reveals a consistent gap between structured prescription records and actual treatment exposure [7–9, 13]. The central finding is that prescriptions alone did not reliably capture when therapy truly starts, how consistently it is used, or whether it is continued at all. Only about one-third of patients had clear note-confirmed initiation near the time of a recorded prescription, while the remainder reflected a mixture of delayed starts, interruptions, “explicit non-initiation,” or lack of documentation (Table 2 and Table S1). This suggests that structured medication orders represent intent to treat rather than confirmed exposure, and that relying on them alone may substantially misclassify real-world treatment patterns.

The heterogeneity in initiation pathways is particularly notable (Table 2 and Table S1; Figure S2, see online supplementary material for a color version of this figure). While most confirmed starts were frictionless, a meaningful proportion of patients initiated therapy only after documented administrative barriers such as insurance or access constraints, or experienced early interruption, often due to gastrointestinal adverse events [14, 15, 24–26]. In addition, a sizable fraction of patients had explicit evidence of non-initiation despite a recorded prescription [16]. Phenotypes with notable barriers also generated more clinical documentation leading to a higher operational burden (Figure S3, see online supplementary material for a color version of this figure; Table S5). Taken together, these findings highlight that access, tolerability, and system-level factors play a central role in shaping real-world uptake of GLP-1 therapies, even in settings where prescribing is common [15, 27].

Differences between note-derived and prescription-based persistence further reinforce this disconnect (Fig. 4). Across both semaglutide and tirzepatide, persistence estimated from structured prescriptions consistently exceeded that derived from clinical notes, with materially different trajectories over time. This divergence likely reflects several factors, including prescriptions that are written but never filled, delays in treatment initiation, discontinuation that is not immediately reflected in medication records, and care fragmentation across health systems. Importantly, note-derived persistence may better approximate true patient-level exposure, as it captures clinician-documented evidence of ongoing use or discontinuation. The magnitude of this gap suggests that studies relying solely on prescription records may overestimate treatment persistence and, by extension, underestimate the real-world effectiveness of these therapies [10–12, 28].

The analysis also provides insight into the reasons that contribute to GLP-1RA discontinuation and the barriers to sustained use. Insurance and cost barriers emerged as leading contributors, alongside perioperative holds and gastrointestinal adverse events. These findings are consistent with known challenges in GLP-1RA therapy but provide direct, large-scale evidence from clinical documentation [14, 15, 27]. The prominence of these factors underscores that real-world effectiveness is shaped not only by pharmacology but also by affordability, access, and tolerability, all of which influence whether patients remain on therapy consistently enough to derive benefit [14, 24, 25].

An additional and underappreciated finding is the early and often concurrent presence of compounded GLP-1 exposure [20, 29]. In more than a third of patients with documented compounding, the first signal occurred before the first structured prescription. This pattern suggests that some patients access therapy outside traditional prescribing workflows and/or that compounded medication use precedes or substitutes for formal prescriptions. The rapid accumulation of compounding exposure early in the treatment course indicates that it may represent an important component of real-world GLP-1 use that is largely invisible in structured data. This has implications for both safety monitoring and effectiveness studies, as compounded formulations may differ in dosing, quality, or adherence patterns.

These findings have important implications for real-world evidence generation. Studies that rely exclusively on structured prescription data may misclassify initiation, overestimate persistence, and fail to capture key dimensions of treatment exposure such as delays, interruptions, and alternative sourcing. Integrating unstructured clinical narratives using scalable approaches such as LLMs enables a more accurate reconstruction of treatment trajectories and provides visibility into the barriers and behaviors that shape real-world use. This is particularly relevant for therapies like GLP-1 receptor agonists, where sustained exposure is closely linked to clinical benefit [28, 30–32]. The technical contribution of this workflow is the integration of LLM-based narrative understanding with deterministic epidemiologic phenotype construction. Traditional clinical NLP systems can extract structured information from clinical notes, but systematic reviews have highlighted persistent challenges in portability, temporal extraction, concept normalization, and extraction of information expressed through heterogeneous clinical context [33, 34]. These limitations are particularly relevant for GLP-1 exposure ascertainment, where clinically meaningful status may appear as active medication use, non-initiation, payer delay, perioperative hold, adverse-effect interruption, outside prescribing, medication reconciliation, or compounded formulation use. Recent LLM studies demonstrate the feasibility of extracting clinical variables from unstructured and semi-structured EHR text using zero-shot or few-shot approaches [17–19]. Our approach extends this paradigm by constraining model outputs to a predefined schema, linking extractions to source evidence, excluding medication-list-only evidence from active-use adjudication, validating extracted events against physician review, and applying deterministic rules to reconstruct patient-level initiation and persistence phenotypes.

Interpretability was operationalized through auditability rather than through attention visualization or token-level attribution. Each extraction was constrained to a predefined schema and linked to source evidence, allowing physician reviewers to inspect the text supporting structural chunk type and medication-status classification. We did not use attention weights or gradient-based token importance as primary explanation methods because prior NLP work has shown that attention weights may not provide faithful explanations of model decisions and that gradient-based explanations can be unstable or manipulable [35, 36]. For clinical extraction, we therefore prioritized source-evidence traceability, physician-adjudicated event-level validation, and deterministic phenotype rules, an approach aligned with recent work emphasizing auditable clinical information extraction [37]. Future work should evaluate whether token-level attribution or semantic attention analyses add incremental value after independent assessment of their stability and faithfulness.

This study has limitations. First, it is a retrospective observational study on EHR data and is thus prone to multiple sources of bias, confounding, incomplete capture, and variation in clinical documentation. The study was descriptive and was not designed to estimate causal effects on clinical outcomes. Second, note-based ascertainment depends on the presence, timing and quality of clinical documentation and may still miss treatment use, interruptions, or compounded exposure that were not explicitly recorded in notes. Third, although the LLM pipeline was evaluated against a physician-adjudicated extracted-event reference standard and showed strong performance for structural chunk triage and medication-status adjudication, residual misclassification remains possible. The inference pipeline was evaluated using a fixed decoding configuration, with temperature 0.1 and top_k = 1, selected a priori to reduce stochastic variability and support schema-constrained output generation. We did not perform a formal sensitivity analysis across alternative temperatures, sampling strategies, prompt variants, or model architectures. Fourth, structured prescription records may be incomplete for medications obtained outside the health system, which could make patients appear to discontinue therapy earlier than they actually did. Fifth, the persistence and compounding analyses were restricted to subsets with sufficient follow-up and documentation density, which may limit extrapolation to the full prescription cohort. Finally, although this study was conducted on a federated EHR network that captures geographically diverse segments of the United States, the findings may not generalize to all populations, care settings, documentation styles, health systems, or populations without local validation. The foundation model may encode biases from its pretraining data, including differential performance across clinical language patterns, documentation styles, and underrepresented patient or care contexts. Pipeline performance may also degrade in environments with different EHR note layouts, medication-list formatting, local abbreviations, specialty documentation practices, prescribing workflows, compounded-medication terminology, or non-US clinical language. External validation and site-specific calibration should therefore be performed before applying this workflow outside the studied federated environment.

In summary, this study demonstrates that real-world GLP-1 therapy exposure is substantially more complex than suggested by structured prescription data alone. Initiation is often delayed or absent despite recorded intent, persistence is overestimated when based solely on prescriptions, and compounded use is common and frequently overlaps with formal prescribing. These findings highlight the need to integrate multiple data sources to accurately capture treatment exposure and provide a more accurate understanding of how GLP-1 therapies are used in routine care.

## Supplementary Material

bpag035_Supplementary_Data

## Data Availability

This study involves the analysis of de-identified Electronic Health Record (EHR) data via the nference nSights Federated Clinical Analytics Platform (nSights). Data shown and reported in this manuscript were extracted from this environment using an established protocol for data extraction, aimed at preserving patient privacy. The data have been de-identified pursuant to an expert determination in accordance with the HIPAA Privacy Rule. Any data beyond what is reported in the manuscript, including but not limited to the raw EHR data, cannot be shared or released due to the parameters of the expert determination to maintain the data de-identification. The corresponding author should be contacted for additional details regarding nSights.
