# Supplementary figures and images for: Auditable large language model curation of clinical notes refines glucagon-like peptide-1 initiation, persistence ascertainment, and compounding use beyond prescription records
Source: Biol Methods Protoc. 2026 Jun 24;11(1):bpag035. doi: 10.1093/biomethods/bpag035 (PMC13353216; doi:10.1093/biomethods/bpag035)

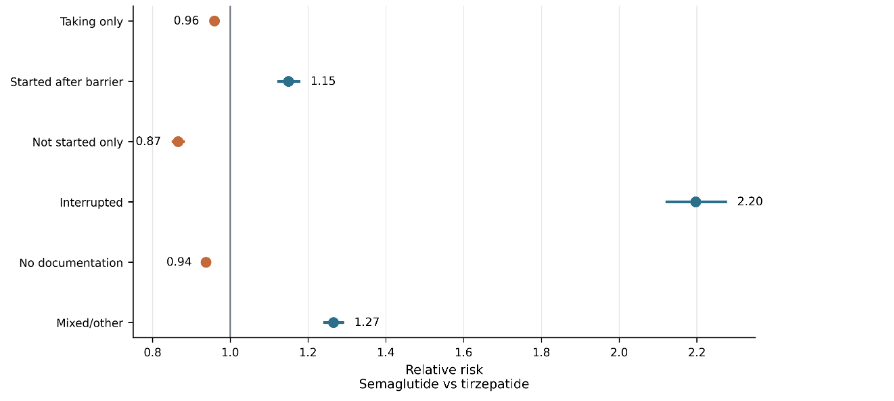

Supplement: bpag035_Supplementary_Data [file bpag035_supplementary_data.zip › Supplementary_Figure_S2.tif]

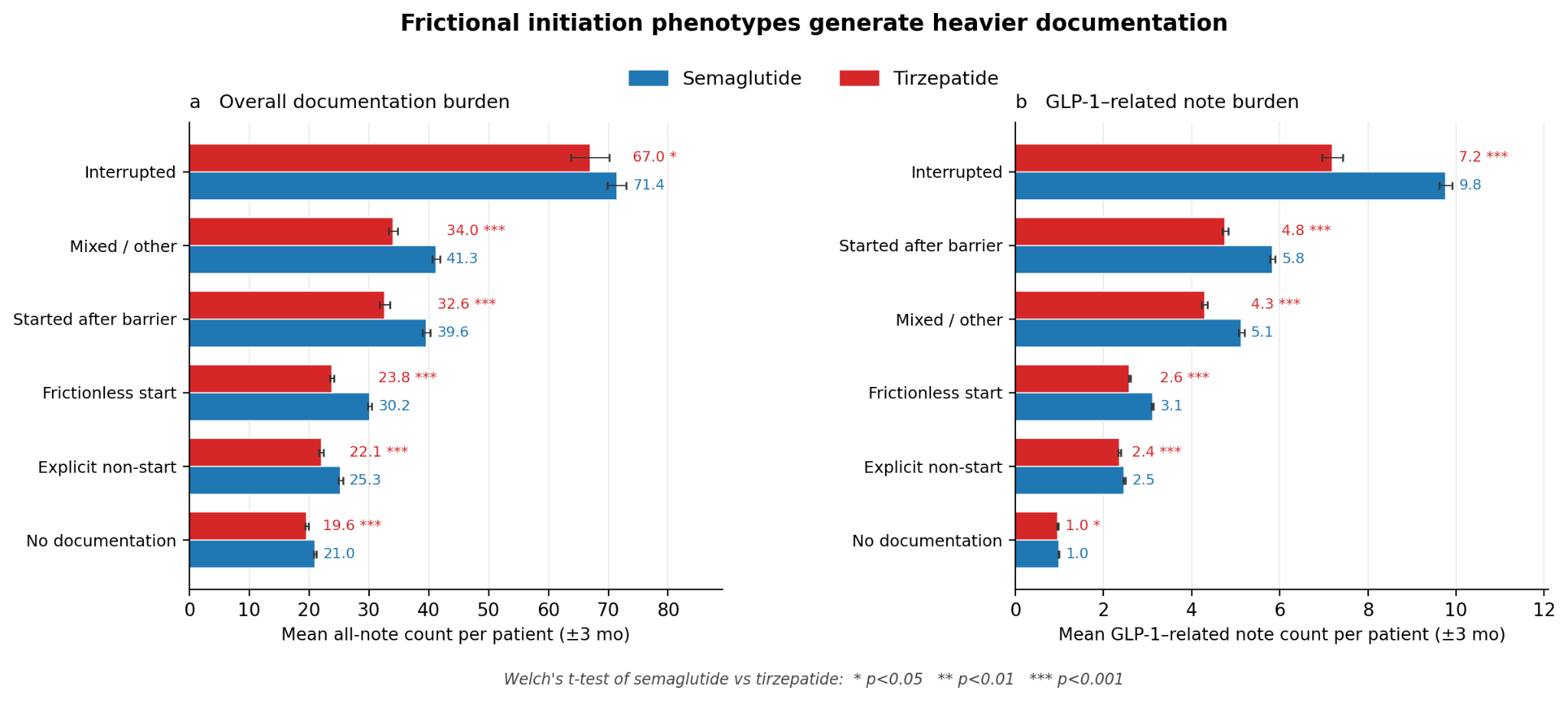

Supplement: bpag035_Supplementary_Data [file bpag035_supplementary_data.zip › Supplementary_Figure_S3.tif]

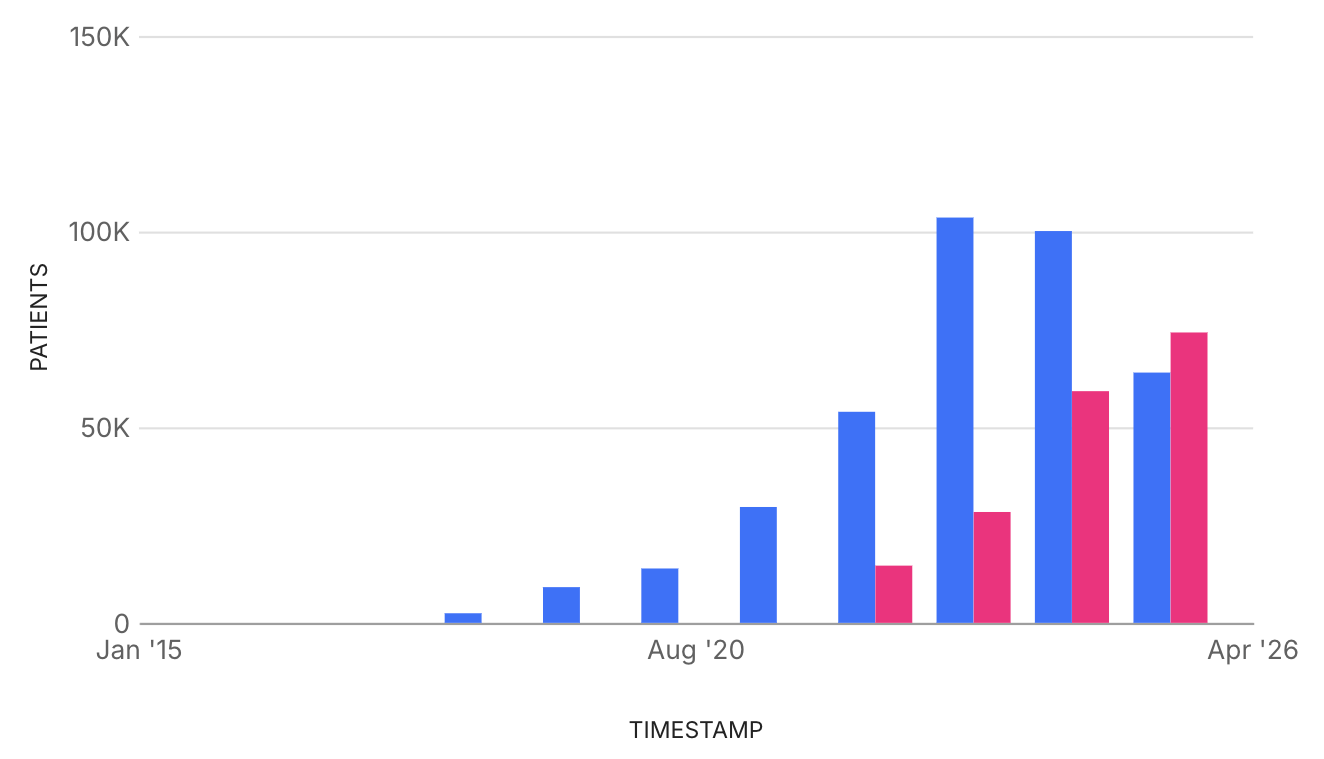

Supplement: bpag035_Supplementary_Data [file bpag035_supplementary_data.zip › Supplementary_Figure_S1.tif]
